# Enhanced Hybrid Vision Transformer with Multi-Scale Feature Integration and Patch Dropping for Facial Expression Recognition

**DOI:** 10.3390/s24134153

**Published:** 2024-06-26

**Authors:** Nianfeng Li, Yongyuan Huang, Zhenyan Wang, Ziyao Fan, Xinyuan Li, Zhiguo Xiao

**Affiliations:** 1 College of Computer Science and Technology, Changchun University, No. 6543, Satellite Road, Changchun 130022, China; 2 School of Computer Science Technology, Beijing Institute of Technology, Beijing 100811, China

**Keywords:** facial expression recognition, lightweight network, attention module, transformer

## Abstract

Convolutional neural networks (CNNs) have made significant progress in the field of facial expression recognition (FER). However, due to challenges such as occlusion, lighting variations, and changes in head pose, facial expression recognition in real-world environments remains highly challenging. At the same time, methods solely based on CNN heavily rely on local spatial features, lack global information, and struggle to balance the relationship between computational complexity and recognition accuracy. Consequently, the CNN-based models still fall short in their ability to address FER adequately. To address these issues, we propose a lightweight facial expression recognition method based on a hybrid vision transformer. This method captures multi-scale facial features through an improved attention module, achieving richer feature integration, enhancing the network’s perception of key facial expression regions, and improving feature extraction capabilities. Additionally, to further enhance the model’s performance, we have designed the patch dropping (PD) module. This module aims to emulate the attention allocation mechanism of the human visual system for local features, guiding the network to focus on the most discriminative features, reducing the influence of irrelevant features, and intuitively lowering computational costs. Extensive experiments demonstrate that our approach significantly outperforms other methods, achieving an accuracy of 86.51% on RAF-DB and nearly 70% on FER2013, with a model size of only 3.64 MB. These results demonstrate that our method provides a new perspective for the field of facial expression recognition.

## 1. Introduction

Facial expressions carry rich emotional information and are vital for humans to express emotions and engage in social activities. Facial expression recognition plays a crucial role in various human-computer interaction systems, such as intelligent question answering, classroom teaching, smart healthcare, crime detection, and fatigue monitoring. It is the most direct and effective way for computers to perceive human emotions, becoming an indispensable component of human–computer interaction [1]. Therefore, people are investing significant efforts to promote the development of facial expression recognition (FER).

The majority of existing FER methods are currently based on convolutional neural networks (CNNs) [2,3,4]. CNN-based methods can achieve excellent performance in controlled environments because the images are typically frontal-facing, with minimal variations in lighting and almost no occlusion. However, when it comes to recognizing unconstrained facial expressions in real-world scenarios, the performance of these methods often falls short of expectations. Therefore, attention-based CNN methods [5,6,7,8] have been proposed. Among them, the region attention network [5] (RAN) can adaptively obtain important regions in occluded and pose-transformed images, addressing issues of occlusion and pose variations in the real world. Li et al. [8] proposed an end-to-end patch gated convolutional neural network, integrating patch-level attention into facial expression recognition to address occlusion. Similar to [5], several methods such as [9,10] employ attention-like mechanisms to focus on the most discriminative facial features, aiming to improve the accuracy of facial expression recognition (FER). However, these methods are constrained by the local receptive field for feature extraction, heavily relying on spatial local features, and the extracted feature maps may not represent complete facial features. To capture global features of images, CNNs break the limitation of local receptive fields by stacking convolutional layers continuously. Yet, in practical applications, especially in resource-constrained environments, this method of increasing network depth to improve recognition accuracy is not practical. Hence, it is challenging to improve the performance of FER using CNN-based models.

In response to the aforementioned issue, this paper proposes a lightweight facial expression recognition method based on a hybrid vision transformer. This method employs MobileViT [11] as the backbone network for feature extraction, utilized to extract both local and global features of facial expressions. At the same time, to address the challenge of recognizing facial expressions in real-world scenarios, we introduce an improved attention module, embedded within the feature extraction network. This module captures facial features at different scales, facilitating the model’s learning of semantic-level feature representations. And through the patch dropping module, complete facial features are reconstructed from incomplete information, compelling the model to learn more robust and discriminative feature representations, while also reducing computational costs. The contributions of this paper are as follows:

1. We propose a lightweight facial expression recognition method based on a hybrid vision transformer. This method integrates an improved efficient multi-scale attention module, enabling the model to capture multi-scale expression image features and enhance the model’s utilization efficiency of key information through cross-dimensional expression feature aggregation. This effectively addresses the occlusion FER problem.
2. We designed a simple and effective patch dropping module, which simulates the non-uniform distribution characteristic of human visual attention, guiding the model to focus on the most discriminative features and reducing the influence of irrelevant features.
3. We conducted extensive experiments on the RAF-DB dataset and the FER2013 dataset. The experimental results demonstrate that our proposed method outperforms mainstream CNN-based FER methods, achieving excellent performance. It is worth noting that our model has only 3.64 MB parameters, which is smaller than most lightweight models and significantly smaller than other transformer-based models.

The subsequent sections of this paper are organized as follows: Section 2 reviews related work on facial expression recognition. Section 3 provides a detailed description of the proposed model. Section 4 conducts experiments to validate the effectiveness of our proposed model. Finally, Section 5 concludes our research work with a summary.

## 2. Related Work

In the past few decades, the field of FER has remained vibrant and has been active in research. In recent years, FER technology has undergone a transition from traditional algorithms to deep learning algorithms, with its core processes typically encompassing three fundamental steps: facial detection, expression feature extraction, and expression classification. In the crucial step of expression feature extraction, traditional methods relied on manually designed feature descriptors, such as Gabor wavelet features [12], local binary patterns (LBP) features [13], and histogram of oriented gradients (HOG) features [14]. These methods based on manual feature extraction often capture limited expression information, exhibiting weak generalization capabilities when handling expression images in complex environmental conditions. The emergence of deep learning technologies in recent years has propelled the development of the facial expression recognition field. CNNs have garnered widespread attention due to their outstanding feature extraction capabilities. They not only extract richer and more complex feature representations from images but also demonstrate robustness under varying image sizes and lighting conditions, ushering in new breakthroughs in facial expression recognition. Although these methods can exhibit rapid and accurate recognition capabilities in controlled experimental environments, the complexity, subtlety, and facial occlusion in real-world application scenarios often limit their recognition performance.

To better recognize facial expressions in real-world scenarios, numerous researchers have further explored and optimized deep learning methods such as CNN in their work. Some researchers solve the occlusion problem in real-life scenes by allowing the model to extract the most discriminative facial expression features. Among them, Cai et al. [15] proposed the island loss function to obtain discriminative expression features by optimizing intra-class compactness and inter-class separation in feature space. Li et al. [7] employed attention mechanisms to guide the network to focus on the most discriminative, non-occluded facial regions. In addition to the methods mentioned above, Pan et al. [16] introduced a novel approach that leverages auxiliary information from non-occluded facial images to enhance the model’s ability to recognize occluded facial expressions. Meanwhile, Wang et al. [5] proposed a region attention network (RAN), which passes regions around facial key points of images to CNN and employs self-attention modules to weigh and fuse the cropped feature vectors for more precise feature representation. Li et al. [17] proposed a novel depth emotion conditional adaptation network (ECAN), which achieves effective alignment of global marginal distributions and end-to-end matching of fine-grained class conditional distributions by fully utilizing low-level label information in the target data, aiming to address the issue of neglected imbalances in emotion category distributions. Yao et al. [18] embedded the HPMI attention module into the VGG-16 network to elevate the importance of critical features, mitigating overfitting issues and enabling accurate recognition of subtle facial expression variations in real-world scenarios. While these deep learning methods enhance the capability of extracting facial expression features by employing larger and deeper networks, they also increase network parameters and computational complexity, thereby reducing network recognition efficiency.

To address the high computational complexity and low recognition efficiency of the aforementioned methods, one of the earliest works in model lightweight was proposed in 2016 by researchers from Berkeley and Stanford, SqueezeNet [19]. In 2017, Google successively introduced three models: MobileNet V1 [20], MobileNet V2 [21], and MobileNet V3 [22], followed by models such as ShuffleNet [23] and ShuffleNet V2 [24] proposed by Megvii. These models exhibit relatively low computational complexity, can be applied to mobile and embedded devices, and achieve good accuracy on the ImageNet dataset. Consequently, some researchers conducted a series of studies based on these lightweight model frameworks. Nan et al. [25] adopted the MobileNet V1 model as the basic framework for feature extraction and introduced attention mechanisms to enhance the model’s ability to extract facial expression features. Han et al. [26] used MobileNet V1 as the basic framework and introduced branch network and attention mechanisms to fully extract inter-class features and intra-class diversity features of facial expressions. Zhang et al. [27] introduced shortcut branches in ShuffleNet V2 to enhance information interaction and feature fusion between channels, and embedded attention modules to enhance the network’s spatial feature extraction capabilities. Additionally, employing other technical means can also achieve model lightweighting. Zhou et al. [28] introduced the frequency multiplication rating uniform rectangular features (URF), leveraging multiplication layers for low-cost frequency domain feature extraction. Thus, the complexity and computational burden of the network have markedly decreased. Cotter et al. [29] proposed the lightweight deep learning model MobiExpressNet, which relies on depth-wise separable convolution to limit complexity and uses rapid downsampling methods to maintain model size.

## 3. Method

We have proposed a lightweight facial expression recognition method based on a hybrid vision transformer, which is an efficient hybrid vision transformer. It includes two key modules: the improved multi-scale attention module and the simple and efficient patch dropping module. In this section, we first introduce the overall architecture of the model, and then we provide detailed descriptions of these two key modules.

### 3.1. The Proposed Architecture

**Figure 1:**
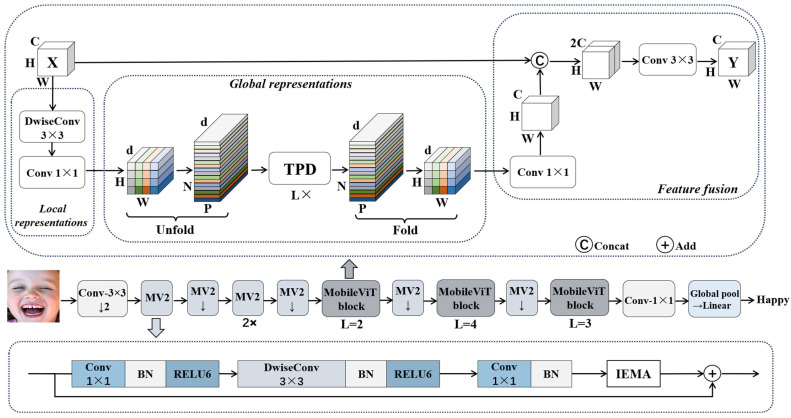
The proposed architecture.

The overall architecture of our proposed model is illustrated in Figure 1. It starts with a 3 × 3 standard convolutional layer, followed by a feature extraction network composed of alternating MobileNet V2 (MV2) and MobileViT blocks. Finally, the classification prediction is obtained through a global pooling layer and a fully connected layer. Afterward, a 1 × 1 convolution is employed to reduce the dimensions of the feature map, alleviating computational burden. Subsequently, the feature maps are fed into the improved efficient multi-scale attention module (IEMA), which enhances the distribution of spatial semantic features, further improving the representational capacity of the features. The MobileViT block consists of three modules: local representation, global representation, and feature fusion. Initially, the feature map undergoes a 3 × 3 convolution to extract local features from the image. Subsequently, a 1 × 1 convolution adjusts the number of channels of the feature map from C to d. Then, the Transformer module with the “Unfold-TPD-Fold” mechanism is employed for global feature extraction, followed by a 1 × 1 convolution to restore the number of channels of the feature map to C. Finally, the feature map is concatenated with the original input image along the channel dimension via a shortcut residual branch, and a 3 × 3 convolution is applied to merge these features to obtain the final global feature output.

In summary, our proposed method adopts MobileViT as the baseline network, and within the base structure of MobileNet V2 (MV2), we introduce IEMA. This module significantly enhances the model’s ability to capture crucial details in facial expressions and effectively addresses occlusion issues. Additionally, we propose a simple and efficient patch dropping (PD) module aimed at further strengthening the model’s ability to handle complex facial expression images. By embedding the PD module into the Transformer, we intuitively reduce computational costs and promote strong interconnections between local blocks in facial expression images, ultimately improving recognition accuracy.

### 3.2. The Improved Efficient Multi-Scale Attention Module

While the baseline network can effectively extract local features of facial expressions, it lacks sufficient integration of information across channels during feature extraction, making it difficult to capture useful feature interactions. To enable the model to learn the correlations between different channels while considering features at different scales, we propose an improved, efficient multi-scale attention module (IEMA). Embedded within the feature extraction network, it aims to aggregate facial features across dimensions, enhancing the model’s efficiency in utilizing key information.

**Figure 2:**
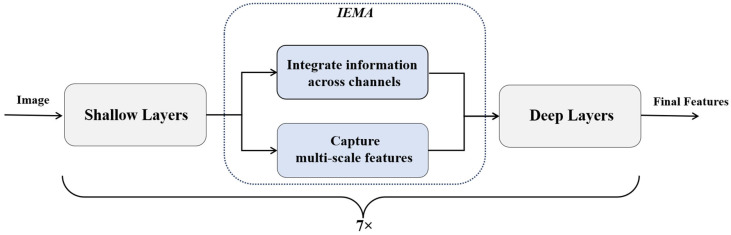
The interaction process between the IEMA module and the backbone network.

During the feature extraction process, the IEMA module interacts with the MobileViT backbone at multiple stages to enhance its feature extraction capability (as shown in Figure 2). Specifically, the input image first passes through the shallow feature extraction part of the backbone network, extracting the initial facial expression features. These initial features then go through the IEMA module, generating multi-scale enhanced features. These enhanced features are then fed back into the backbone network, which further processes them through its deeper layers to extract higher-level features. This process is repeated, with a total of 7 IEMA modules applied at different stages of feature extraction. Ultimately, through this iterative interaction, the feature extraction capability of the backbone network is significantly enhanced, resulting in more accurate facial expression features.

**Figure 3:**
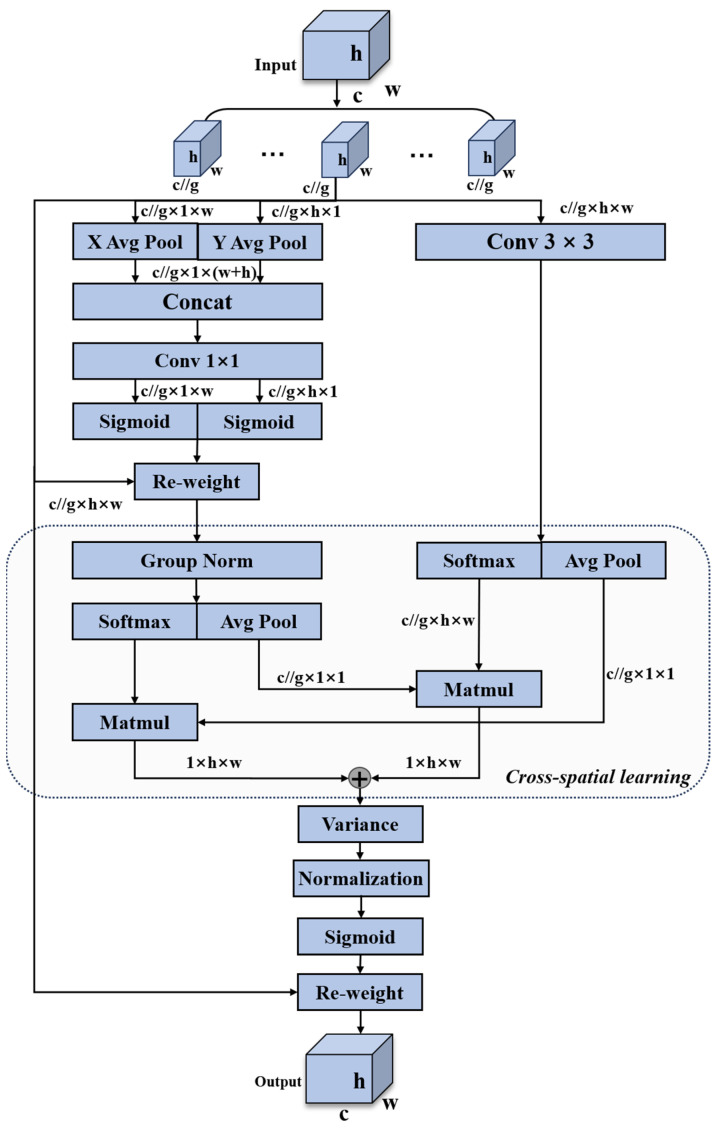
The schematic diagram of the improved efficient multi-scale attention module structure.

The IEMA adopts a parallel structure to process input data, enabling the model to train faster and handle features at different scales to improve recognition accuracy. It divides the features evenly into multiple sub-features in the channel dimension, ensuring a balanced distribution of facial expression information within each feature group while retaining the complete information of each channel. We integrate information across dimensions through feature aggregation to capture useful feature interactions between different channels. Finally, by dynamically adjusting the weights of different positions in the feature maps, we highlight important features while suppressing irrelevant ones, enhancing the model’s efficiency in utilizing key information. This enables the IEMA to effectively handle facial expression recognition in the presence of occluded images. The schematic diagram of the IEMA module structure is shown in Figure 3.

In the IEMA module, three parallel pathways are utilized to extract attention weight descriptors for grouped feature maps. Two of these pathways are located within the 1 × 1 branch, while the third parallel pathway is within the 3 × 3 branch. The 1 × 1 branch employs two 1D global average pooling operations along different spatial directions to encode channels and aggregates attention maps from both directions through multiplication to achieve cross-channel interaction. Simultaneously, the 3 × 3 branch captures local cross-channel interactions via a 3 × 3 convolutional kernel to expand feature representation and capture multi-scale features. This approach effectively combines global and local features, enhancing the model’s ability to capture features at different scales. Thus, the IEMA module encodes information between channels to adjust the importance of different channels and preserves precise spatial structural information within channels.

Specifically, the input features are first divided into *G* sub-features along the cross-channel dimension direction for learning different semantics, as shown in Equation (1)

(1) $$X=\left[X_{0},X_{i},\ldots ,X_{G-1}\right],X_{i}\in R^{C//G\times H\times W}$$

The subsequent *G* sub-features enter the parallel subnetwork composed of 1 × 1 and 3 × 3 branches to extract weight descriptors of the feature maps. The 1 × 1 branch performs global average pooling on the input feature maps along the width and height directions separately to obtain feature maps in both width and height dimensions. Specifically, after the average pooling along the width direction, the feature $X\in R^{C//G\times H\times 1}$ is obtained, and the feature is mapped onto the height dimension. Following the average pooling along the height direction, the feature $X\in R^{C//G\times b\times W}$ is obtained, and the feature is mapped onto the width dimension. Then, the feature maps from these two parallel stages are merged and transposed to the same dimension for stacking, thus combining the height and width features to form a feature map $X\in R^{C//G\times 1\times (H+W)}$. Subsequently, to achieve different inter-channel interaction features between the two parallel paths in the 1 × 1 branch, we aggregate the attention features of two channels within each group simply by multiplication. Then, the sigmoid function is applied to obtain attention states for the width and height dimensions, respectively. In the 3 × 3 branch, a 3 × 3 convolution kernel is used to capture local inter-channel interaction to expand the feature space. Finally, to achieve richer feature aggregation, we propose a cross-space information aggregation method. A tensor is introduced in each of the 1 × 1 and 3 × 3 branches, and global information is encoded using 2D global average pooling operations, encoding the global information into the output of the 1 × 1 branch, denoted as $R_{1}^{1\times C//G}\times R_{3}^{C//G\times HW}$. The operation of 2D global average pooling is shown in Equation (2).

(2) $$z_{c}=\frac{1}{H\times W}\sum_{j}\sum_{i}^{W}x_{c}(i,j)$$

Softmax, a non-linear function, is applied at the output of the 2D global average pooling to enhance computational efficiency and fit the linear transformation. The first spatial attention map is derived by performing element-wise multiplication of the outputs from the aforementioned parallel processing with matrix dot product operations, which aggregates spatial information at different scales within the same processing stage. Additionally, we encode global spatial information in the 3 × 3 branch using 2D global average pooling. The output feature map within each group is calculated as the aggregation of the two generated spatial attention weight values. Subsequently, by calculating the spatial average of the feature map in each channel, we obtain a value representing the overall level of that channel. Next, for each pixel position within each channel, we calculate the square of the difference between its value and the corresponding channel mean, measuring the deviation of each pixel value from the channel mean. This guides the model to focus on the features in the image, enhancing the efficiency of utilizing key information. Then, normalization is performed, and finally, attention weights are formed through the sigmoid function to obtain the output features.

### 3.3. Patch Dropping Module

The human visual system does not uniformly attend to the entire visual field when processing visual information; instead, it tends to focus attention on key visual elements. This attention allocation mechanism enables humans to rapidly identify and respond to crucial visual information in complex scenes. Inspired by this mechanism, we believe that there are critical local regions in the feature maps of facial expressions that play a decisive role in expression recognition. Therefore, we propose the patch dropping module, aiming to mimic the attention allocation mechanism of the human visual system towards local features.

The patch dropping module enhances the robustness of the feature extraction process by introducing noise. Specifically, randomly dropping patches in the feature map introduces perturbations that force the model to learn feature representations without relying heavily on specific local regions, but instead gaining a more comprehensive understanding of the entire feature map. This helps the model generalize better when faced with different input images. Additionally, regularization is a technique in deep learning to prevent overfitting, with dropout being a commonly used method. Dropout reduces the model’s dependence on specific neurons by randomly dropping them, thereby improving generalization. Patch dropping applies a similar concept at the level of feature patches, which means it affects the model’s feature extraction process at a higher level, enabling the model to learn more global and robust feature representations. From the perspective of ensemble learning, each operation of dropping feature patches can be seen as training different sub-models. By continuously changing which feature patches are dropped during training, the model effectively learns multiple distinct sub-models. This approach enhances the model’s robustness and generalization capabilities because the model needs to perform well across different combinations of features.

**Figure 4:**
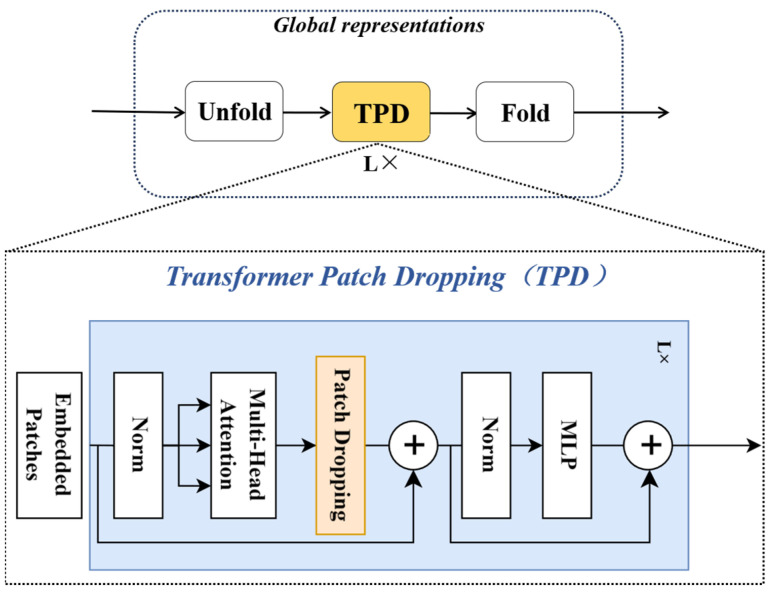
The interaction process between the patch dropping module and the backbone network.

During the feature extraction process, the image first undergoes preliminary feature extraction through several MV2 modules in the backbone network. Each MV2 module processes the input image using depthwise convolution, resulting in an initial facial expression feature map. Before entering the patch dropping module, the feature map is converted into multiple patches through an unfold operation. These patches are equal-sized local regions, each representing a portion of the feature map. The patch dropping module randomly drops certain patches. This process introduces noise, preventing the model from relying on specific local regions and thereby promoting the learning of more comprehensive feature representations. The feature map, after having patches dropped, contains randomly missing local regions, forcing the model to extract more information from the remaining patches. The feature map processed by the patch dropping module is then restored to its original size through a fold operation and re-enters the backbone network. The interaction process is as shown in Figure 4.

**Figure 5:**
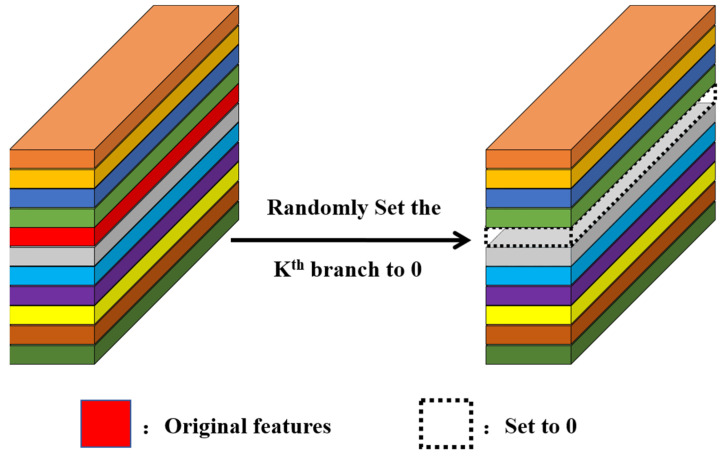
Patch dropping module.

The design of this module (as shown in Figure 5) aims to enhance model performance in the following ways:

1. Randomization of Local Features: By randomly dropping patches, the model is encouraged to explore different regions in the feature map, thereby enhancing sensitivity to local features.
2. Enhancement of Model Generalization: As the model encounters different combinations of patches during training, it helps the model learn more generalized feature representations, reducing reliance on specific features.
3. Facilitation of Multi-Scale Feature Learning: Patch dropping encourages the model to focus on features at both large and small scales, as randomly dropping patches may compel the model to extract more information from the remaining patches.
4. Synergy with Multi-Head Self-Attention Mechanism (MSA): MSA allows the model to consider the relationships between multiple patches simultaneously. Even if certain patches are dropped, MSA can still help the model maintain an understanding of the entire feature map. This synergy enables the model to maintain strong facial expression recognition capabilities even in the absence of local information.

Specifically, the model generates feature maps through convolutional layers, which contain crucial information about facial expressions. These feature maps are then divided into multiple equally sized patches, which serve as inputs to the MSA mechanism. We use a set of feature maps as input and treat each feature map as a whole. During training, a feature map is randomly selected from a uniform distribution, and all its elements are zeroed out based on a Bernoulli distribution. This zeroing-out probability follows a Bernoulli distribution, and the feature map is no longer activated in subsequent layers. This design is similar to the gradient stopping mechanism of Dropout, which guides the MSA mechanism to explore diverse and discriminative facial regions.

In each training iteration, for each sample and each MSA within each MobileVit block, and for each of the k self-attention (SA) operations within the MSA, one SA operation is randomly chosen. This selection process is independent, meaning each MSA can choose a different SA operation. This introduces necessary randomness to samples in the transformer and different MSA blocks, enhancing the model’s generalization ability. Similar to dropout, PD is activated only during training and deactivated during inference. Additionally, unlike dropout, PD does not require weight rescaling after fully connected layers. Through this strategy, the model is incentivized to learn more useful information during training, while the collaborative work of multiple self-attention layers further strengthens the model’s expressive power.

## 4. Experimental Results

In this section, we first introduce the experimental environment, datasets, and preprocessing of training data. Finally, we analyze and discuss the experimental results, comparing them with other mainstream and excellent algorithms.

### 4.1. Preparation for Experiment

Experimental environment: In this experiment, we built the model based on the PyTorch deep learning framework. To accelerate the model’s convergence, we utilized CUDA for GPU-accelerated computing. For hardware configuration, we utilized the Nvidia GeForce RTX 3090 GPU with 24 GB of memory to ensure efficient experiment execution. The experimental parameter settings are as follows: optimizer: AdamW, learning rate: 0.0002, epochs: 200, batch size: 8.

Pre-training: To leverage existing knowledge transfer and alleviate the training burden, the model in this study initializes with weights pretrained on the ImageNet dataset during the initialization stage. This strategy is based on the principles of transfer learning, enabling the model to learn rich, general features from the large-scale ImageNet dataset. Consequently, it accelerates the convergence process and improves the generalization capability of the final model.

**Figure 6:**
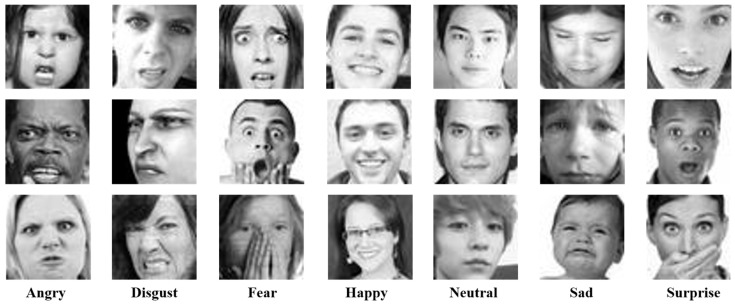
FER2013 dataset samples.

FER2013: The FER2013 dataset [30], created by Goodfellow et al., was used for the ICML 2013 facial expression recognition challenge. This dataset consists of 48 × 48 grayscale facial expression images. The FER2013 dataset comprises facial expression data from real-life natural faces, including individuals of different nationalities, skin colors, and ages ranging from 0 to 75 years old. Due to the presence of non-facial data and false labels in FER2013, the recognition rate of facial expressions is relatively low. Figure 6 shows some samples of facial expressions from this dataset, with some facial images not undergoing center alignment processing. This requires the designed network model to have good robustness and generalization ability, reflecting the representation capability of the network model from the side. Table 1 illustrates the number of facial expressions contained in the FER2013 dataset and the number of each expression category.

**Figure 7:**
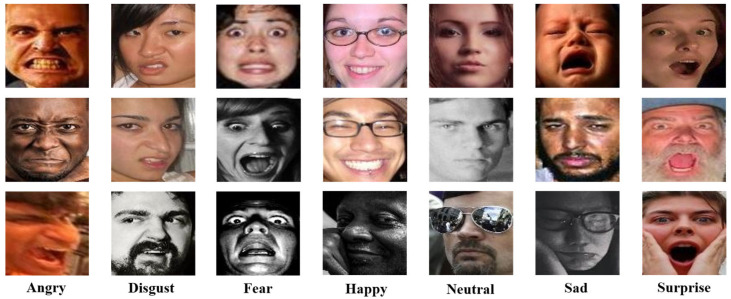
RAF-DB dataset samples.

RAF-DB: RAF-DB [31] is a real-world facial expression database used for facial expression recognition research. This dataset contains facial images captured in non-controlled environments, providing facial expression samples that are closer to real-world application scenarios. It includes 29,672 real-world facial images, independently labeled by approximately 40 trained human workers. In our experiment, we used the provided single-label subset, which includes 15,339 facial expression images, consisting of six basic expressions (happiness, surprise, sadness, anger, disgust, and fear) and neutral expressions. Figure 7 shows some samples of facial expressions from this dataset. Table 1 illustrates the number of facial expressions contained in the RAF-DB dataset and the number of each expression category.

### 4.2. Ablation Experiments

To facilitate a scientifically rigorous analysis of the experimental results, we conducted ablation experiments on the proposed model. By gradually removing certain parts of the network model, we systematically observed the model’s performance on the RAF-DB and FER2013 datasets to understand how these changes affect the predictive performance. As we separately integrated improved efficient multi-scale attention modules and patch dropping modules into the baseline network, we will conduct ablation studies on both. In our experiments, we used MobileViT pretrained on the ImageNet-1K dataset as the baseline network to validate the effectiveness of our proposed model. All experiments were conducted using the same experimental setup. The experimental results are shown in Table 2.

From Table 2, it can be observed that the baseline network MobileVit exhibits a high accuracy in the task of facial expression recognition, thereby clearly demonstrating the excellent performance of our proposed model. On the RAF-DB dataset, compared to the baseline network, the addition of the improved efficient multi-scale attention module to the baseline network resulted in an increase in recognition accuracy of nearly 1%. This is because the RAF-DB dataset comprises images of males and females of various ethnicities and ages worldwide, with significant inter-class feature differences. The improved efficient multi-scale attention module can extract discriminative facial expression details, thereby significantly improving recognition performance. The addition of the patch dropping module to the baseline network led to high similarity in intra-class features while learning potential discriminative features affecting facial recognition. This resulted in the recognition accuracy of the baseline network with the improved efficient multi-scale attention module being superior to that of the baseline network with the patch dropping module. Our proposed model combines the advantages of the improved efficient multi-scale attention module and the patch dropping module, leading to a significant improvement in recognition accuracy.

In Table 2, the model’s recognition accuracy on the FER2013 dataset is lower than that on the RAF-DB dataset, which is due to inherent issues with the FER2013 dataset itself, leading to suboptimal recognition performance compared to the RAF-DB dataset. The addition of the patch dropping module to the baseline network on the FER2013 dataset comprehensively focused on the interaction among local blocks of facial expression feature maps, learning more about the relationships between facial expression features, and expanding the learnable range of intra-class features. Consequently, the improvement was more pronounced compared to the baseline network with the improved efficient multi-scale attention module. Overall, our proposed model, which combines the advantages of the improved efficient multi-scale attention module and the patch dropping module, exhibits a more noticeable overall improvement on the FER2013 dataset.

In conclusion, our proposed method achieves a significant improvement in recognition performance on both the RAF-DB and FER2013 datasets without increasing the size of the base network model.

### 4.3. Comparison with Other Methods

**Figure 8:**
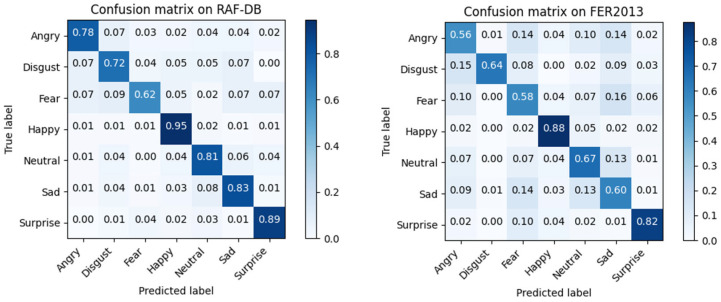
Confusion matrix on RAF-DB (**left**) and FER2013 (**right**).

To more accurately investigate the recognition performance of our model for various facial expressions, we trained separate models on the RAF-DB and FER2013 datasets and generated confusion matrices for analysis, as shown in Figure 8.

From the obtained confusion matrices, it can be observed that our model achieves the highest recognition accuracy for the “happy” expression on both datasets, reaching 95% and 88%, respectively. This is because the “happy” expression has more distinguishable features, such as raised corners of the mouth and wrinkles around the eyes, making it easier to differentiate from other expressions. Moreover, the “happy” expression has the largest number of samples in both datasets, which facilitates the learning process of the model. For the RAF-DB dataset, the expressions “surprise”, “neutral”, “sad”, and “anger” also achieve relatively high recognition accuracy. Additionally, the “surprise” expression features a widened mouth and widened eyes, while the “fear” expression also exhibits a widened mouth, but with greater magnitude, making it prone to confusion with “surprise”. Due to similar features such as furrowed brows and wrinkled forehead, “disgust” is easily confused with “sad”. Compared to the RAF-DB dataset, the model’s recognition accuracy for negative expressions such as “fear” and “anger” is lower on the FER2013 dataset. This is because these expressions inherently share strong similarities, with subtle variations in facial features such as downturned mouth corners and furrowed brows. These similarities make it difficult for human eyes to fully distinguish between them, posing a challenge for the recognition task. Additionally, due to the presence of incorrect labels and a portion of samples not being front-facing or containing obscured expressions, the FER2013 dataset itself poses greater challenges in terms of scale and recognition difficulty compared to other facial expression datasets like RAF-DB.

**Figure 9:**
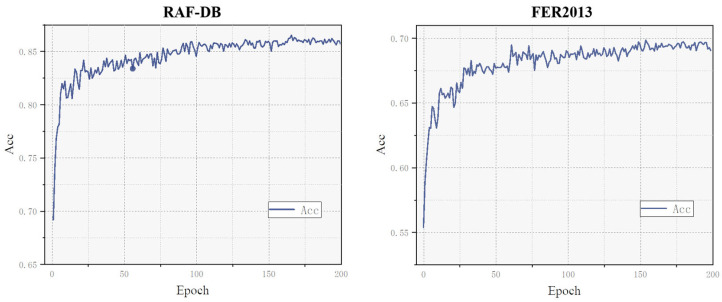
Training curves on RAF-DB (**left**) and FER2013 (**right**).

The experimental training process of the proposed method is illustrated in Figure 9. On the RAF-DB dataset, the model’s recognition accuracy grows slowly around the 100th epoch, and by the 160th epoch, the accuracy gradually stabilizes, reaching a peak accuracy of 86.51%. On the FER2013 dataset, the model’s recognition accuracy grows slowly around the 100th epoch, and by the 150th epoch, the accuracy gradually stabilizes, reaching a peak accuracy of 69.84%. Combining the results from both datasets, it is evident that this model can achieve robust performance across different datasets, especially showing high accuracy and stability on the RAF-DB dataset. Although the accuracy on the FER2013 dataset is slightly lower than RAF-DB, considering the potential complexity of the FER2013 data, this still demonstrates the model’s good generalization ability.

To demonstrate the effectiveness of our proposed model in facial expression recognition tasks, comparative experiments were conducted with ResNet18, ResNet50, VGG16, VGG19, and AlexNet in terms of model parameters and recognition accuracy. The experimental results are presented in Table 3. On the RAF-DB dataset, our proposed method achieved the highest accuracy among all models, demonstrating outstanding performance. Compared to the classic ResNet18 model, the accuracy was improved by 2.41%. Additionally, on the FER2013 dataset, the recognition accuracy of our method reached 69.84%, surpassing the average performance of these methods. These results not only validate the effectiveness of our method but also demonstrate its excellent generalization ability. In terms of model size, the model proposed in this paper is only 3.64 MB, which is significantly smaller compared to current mainstream algorithms. Overall, our proposed method achieves high recognition accuracy while implementing a lightweight model, demonstrating its efficiency and superiority.

To further demonstrate the effectiveness of our proposed method, we conducted comparative experiments with existing state-of-the-art methods on the FER2013 and RAF-DB datasets. Advanced methods mainly include recent innovative approaches such as DCN, InceptionV4, and lightweight networks like Mini-Xception and the MobileNet series. Additionally, classification network models incorporating attention mechanisms and transformer-based network models are also included. The experimental results are shown in Table 4 and Table 5.

From the experimental results, it can be observed that for the RAF-DB dataset, the recognition accuracy of advanced methods is above 67%, while our method achieves an accuracy of 86.51%. Furthermore, among the MobileNet series models, MobileNetV1 achieves the highest recognition accuracy. However, compared to MobileNetV1, our model achieves a 4.89% improvement in recognition accuracy with only a 0.44 MB increase in parameter size. This further demonstrates that our method can ensure recognition accuracy under lightweight implementation conditions. For the FER2013 dataset, although the overall recognition accuracy is slightly lower than that of the RAF-DB dataset, the accuracy of mainstream models still remains above 66%. Our method still achieves an accuracy of 69.84%, surpassing the average level. In terms of model parameter count, although our method is slightly higher than MobileNetV2 and MobileNetV3-small, considering its significant improvement in accuracy, this slight increase in parameter count is entirely reasonable.

In summary, our proposed method has demonstrated outstanding performance on both the RAF-DB and FER2013 datasets. It not only surpasses multiple existing models in terms of accuracy but also achieves this feat while keeping the model lightweight. These results thoroughly validate the efficiency and superiority of our method, indicating its potential and practical value in the field of facial expression recognition.

To demonstrate that our method has far fewer parameters compared to transformer-based methods, Table 6 compares the total parameters of our model with existing transformer-based facial expression recognition methods. The comparison results show that our model size is 92.97%, 92.86%, 87.14%, and 87.45% smaller than those of [42,43,44,45], respectively.

## 5. Conclusions

In this paper, we introduce a lightweight facial expression recognition method based on a hybrid vision transformer. Our method involves an improved efficient multi-scale attention module, designed to capture multi-scale facial features, integrate information across different channels, and capture useful feature interactions. This facilitates richer feature integration, enabling the model to more effectively capture key facial expression regions, thereby significantly improving classification performance. Furthermore, we proposed a simple and efficient patch dropping structure, which mimics the non-uniform distribution characteristic of human visual attention. It guides the model to focus on the most discriminative features and reduce the influence of irrelevant features. This not only enriches the detailed information of expressions but also enhances the overall feature extraction capability of the model. Through a series of ablation experiments, we validated the effectiveness of the proposed modules and compared them with state-of-the-art facial expression recognition algorithms, confirming the superiority of our model. Compared to other lightweight models, our model demonstrates excellent recognition performance while maintaining a low parameter count. Our model has significantly fewer parameters than transformer-based facial expression recognition methods, highlighting the advantage of being lightweight.

Considering that the proposed model has shown significant improvements in facial expression recognition, there are still certain limitations that need to be addressed in future work. In real-world scenarios, faces are often partially obscured by objects such as glasses, masks, or hands, and different lighting conditions can affect the visibility of facial features. Future research should focus on enhancing the model’s performance under these challenging conditions. Possible approaches include integrating data-augmentation techniques or utilizing additional datasets. Additionally, we will pay more attention to the model’s generalization ability across different demographic groups, including various age groups, genders, and ethnicities, to ensure that the model performs equally well across diverse populations, which is crucial for its practical application. In future experiments, we will collect and incorporate more diverse datasets to evaluate and improve the model’s fairness and generalization. To prevent biased results caused by training bias, we have already taken initial steps by using regularization and data augmentation techniques. However, further work is needed to refine these measures. Techniques such as bias detection and correction algorithms will be included in our future research.

Moreover, we will focus on integrating multiple modalities, including facial expressions, voice, physiological signals, and other information sources, to explore methods for improving facial expression recognition accuracy under occlusion conditions and enhancing performance in complex environments. By addressing these limitations, we aim to design a more robust, fair, and generalizable facial expression recognition system that is suitable for real-world applications.

## Figures and Tables

**Table 1:** Number of different expressions in the RAF-DB and FER2013 datasets.

Class	Angry	Disgust	Fear	Happy	Neutral	Sad	Suprise
RAF-DB	867	877	355	5957	3204	2460	1619
FER2013	4953	547	5121	8989	6198	6077	4002

**Table 2:** Ablation experimental research on key modules of our model. In the table, √ indicates that the module is included in the model.

The Improved Efficient Multi-Scale Attention Module	Patch Dropping Module	Accuracy (%)		Parameters (MB)
		RAF-DB	FER2013	
		84.63	68.27	3.64
√		85.61	69.16	3.64
	√	85.25	69.49	3.64
√	√	86.51	69.84	3.64

**Table 3:** Comparative experimental results with mainstream CNN algorithms.

Model	RAF-DB (%)	FER2013 (%)	Parameter (MB)
ResNet18 [32]	84.10	70.09	11.69
ResNet50 [32]	86.01	71.26	25.56
VGG16 [33]	81.68	68.89	14.75
VGG19 [33]	81.17	68.53	20.06
AlexNet [34]	55.60	67.51	60.92
Ours	86.51	69.84	3.64

**Table 4:** Recognition performance of each model on the RAF-DB dataset.

Model	Accuracy (%)	Parameters (MB)
Capsule-based Net [35]	77.78	-
DBA-Net(DenseNet-161) [36]	79.37	42.9
PG-CNN [8]	82.27	-
DLP-CNN [37]	84.13	-
Mean+ASL+L2SL [38]	84.69	-
gACNN [6]	85.07	>134.29
Mini-Xception	76.26	-
MobileNetV1 [20]	81.62	3.2
MobileNetV2 [21	67.77	2.3
MobileNetV3-small [22]	68.29	1.5
ViT [39]	83.44	86
Vit-TL [40]	84.25	>86
CVT [41]	82.27	51.80
FER-VT [42]	84.31	-
Ours	86.51	3.64

**Table 5:** Recognition performance of each model on the FER2013 dataset.

Model	Accuracy (%)	Parameters (MB)
Inception V4 [43]	66.80	**-**
E-FCNN [44]	66.17	-
DCN [45]	69.30	-
MobileNetV2 [21]	67.90	2.3
MobileNetV3-small [22]	67.50	1.5
Region ViT [46]	56.03	40.8
Tokens-to-Token ViT [47]	61.28	21.5
Deep ViT [48]	43.45	55
CrossViT [49]	50.27	43.3
Ours	69.84	3.64

**Table 6:** Comparison of parameters.

Model	Year	Parameters (MB)
CVT [41]	IEEE Trans 2021	51.80
D-DW-Cov.-T [50]	ICLR 2022	51
Swin-T [51]	ICCV 2021	28.3
ELSA-Swin-T [52]	2021	29
Ours		3.64

## Data Availability

The sources of the datasets in this article are as follows: FER2013: https://www.kaggle.com/c/challenges-in-representation-learning-facial-expression-recognition-challenge (accessed on 16 November 2023); RAF-DB: http://www.whdeng.cn/RAF/model1.html (accessed on 28 December 2023). Readers can apply based on the above URL.
